# Mechanisms of Microorganisms Alleviating Drought and Salt Stresses in Plants

**DOI:** 10.3390/microorganisms13112565

**Published:** 2025-11-10

**Authors:** Di Feng, Wenxiang Li, Pengfei Huang, Meiying Gu, Guangmu Tang, Yanhong Ding, Gang Cao, Wanli Xu

**Affiliations:** 1 Key Laboratory of Saline–Alkali Soil Improvement and Utilization (Saline–Alkali Land in Arid and Semi-Arid Regions), Ministry of Agriculture and Rural Affairs of the People’s Republic of China, Xinjiang Academy of Agricultural Sciences, Urumchi 830091, China; 2 Farmland Irrigation Research Institute, Chinese Academy of Agricultural Sciences, Xinxiang 453003, China

**Keywords:** abiotic stress, drought stress, salt stress, endophytes, rhizosphere microorganisms, plant–microbe interactions

## Abstract

Drought and salt stresses are critical environmental constraints affecting plant growth and development, and microorganisms can enhance plant tolerance to these abiotic stresses through complex mechanisms. This review systematically synthesizes the core mechanisms by which microorganisms regulate plant physiological and biochemical processes under such stresses, specifically including the following: (1) regulating the perception and transduction of abiotic stress signals to enhance plant adaptive responses; (2) boosting gene expression and protein synthesis for overall plant metabolic regulation; (3) activating the antioxidant system to strengthen plant tolerance; (4) modulating plant hormone levels to stimulate growth in response to adversity; (5) enhancing plant nutrition and absorption to improve resilience; (6) optimizing the photosynthesis system to promote the synthesis of essential substances, safeguarding plant growth and development amidst adversity. Finally, the application of microbial inoculants in saline–alkali soil improvement and crop cultivation in arid areas and prospective research directions are discussed.

## 1. Introduction

Within the heterogeneous landscape of abiotic stresses, drought and salt stresses are preeminent constraints that severely impair plant vitality. They trigger physiological and biochemical disruptions, such as enzyme dysfunction, metabolic imbalance and altered hormone signaling [1]. These disruptions further disrupt plant growth and development, leading to reduced germination, diminished vigor, yield losses or even death. Beyond biological harm, these stresses also cause substantial agricultural economic losses and undermine ecological balance [1,2]. To maintain dynamic homeostasis under stress, plants have evolved intrinsic regulatory mechanisms: they alleviate or counteract stress-induced damage by activating antioxidant systems, initiating osmotic protection mechanisms, and inducing the expression of stress-tolerant proteins [3,4]. However, abiotic stress forces plants into a growth–defense trade-off—they sacrifice growth rate, yield, or quality to prioritize stress defense [5]. As stress intensity escalates, the plant’s regulatory system fails to sustain the balance between growth and defense, ultimately leading to severe or irreversible impairment of growth and development processes.

Beneficial microorganisms can enhance plant abiotic stress resistance by establishing interactions with plants—either endophytically (colonizing internal plant tissues) or rhizospherically (associating with root surfaces) [6]. Based on their colonization niche, plant-associated microorganisms are broadly categorized into endophytes and rhizosphere microorganisms [7,8]. Endophytes exhibit localized or systemic colonization of plant organs (e.g., roots, stems, leaves, fruits, rhizomes); for instance, endophytic fungi extend hyphae through plant intercellular spaces to acquire nutrients and carbohydrates from the apoplast [9]. Beneficial endophytes enhance plant growth and abiotic stress tolerance via multiple pathways, including improving nutrient and water uptake, increasing water use efficiency, regulating endogenous hormone levels, and boosting viability competitiveness [10,11,12].

Rhizosphere microorganisms, by contrast, are tightly attached to rhizosphere soil particles. This niche is defined by a mutualistic “inter-utilization” relationship: plant roots and their exudates provide nutrients and energy for rhizosphere microbes, while microbes reciprocate by producing plant-growth-promoting substances or degrading phytotoxic compounds [13].

Prior reviews have explored microbial roles in plant stress tolerance, but critical gaps remain. Feng et al. summarized mechanisms by which exogenous substances (including chemicals and microorganisms) alleviate plant salt and drought stress, proposing six common pathways—inducing osmolyte synthesis, activating antioxidant enzymes, regulating hormone production, mediating gene expression and signal transduction, improving photochemical systems, and microbial regulation [14,15]. However, their coverage of microbial regulatory roles was limited and unsystematic. Kumar et al. (2020) focused on plant-growth-promoting bacteria (PGPB) and the phytomicrobiome, reviewing their roles in mitigating plant salt stress via phytohormone regulation, extracellular polymeric substance (EPS) production, and osmolyte synthesis with antioxidant activity induction [16]. For rhizosphere microorganisms, Braud et al. (2009) and Hayat et al. (2010) classified plant-growth-promoting rhizobacteria (PGPR)-mediated abiotic stress tolerance into direct pathways (e.g., promoting nutrient absorption) and indirect pathways (e.g., inducing systemic tolerance via microbial metabolites) [17,18]. Meanwhile, Gupta et al. (2022) reviewed plant associations with PGPR in mediating plant drought and salt tolerance mechanisms, including the core defense network of reactive oxygen species (ROS) balance, regulation pathways of osmotic and ion homeostasis, and expression [19].

In summary, existing studies focus on either drought or salt stress, or on one microbial group at a time, with limited integration of microbial regulatory pathways across both stress types.

Given the rapid advancement of this field, a comprehensive, timely synthesis is needed to foster a systematic understanding of plant–microbe interactions, as well as the functional relationships between endophytes and rhizosphere microorganisms. This review aims to fill this gap by synthesizing current research on microbial involvement in regulating plant stress tolerance mechanisms in response to both drought and salt stress.

## 2. Plant Regulatory Pathways in Response to Drought and Salt Stresses

Drought and salt stresses disrupt plant cellular homeostasis via osmotic imbalance, ionic toxicity, and oxidative damage, driving plants to activate intrinsic regulatory pathways for survival—these pathways form the core targets for microbial intervention, mainly including three interconnected modules [3,4].

### 2.1. Stress Signal Perception and Transduction

Plants sense stress via membrane-localized sensors. For drought, plasma membrane aquaporins and osmosensors (e.g., *Arabidopsis* AtHK1) detect changes in turgor pressure/osmolarity, triggering the mitogen-activated protein kinase/calcium-dependent protein kinase (MAPK/CDPK) phosphorylation cascade [20,21]. For salt, Salt Overly Sensitive 1 (SOS1) transporters (Na^+^ extruders) interact with SOS3 calcium sensors to activate the SOS pathway [21]. Abscisic acid (ABA) amplifies signals: stress-induced ABA binds PYR/PYL/RCAR receptors, inhibiting protein phosphatases 2C (PP2Cs) and activating sucrose non-fermenting-1-related protein kinases 2 (SnRK2s), which in turn regulate stomatal closure and ion homeostasis [20].

### 2.2. Stress-Responsive Gene Expression Regulation

Plants reprogram transcriptomes via transcription factors (TFs). DREB/CBF family (e.g., DREB2A) binds DRE/CRT elements to upregulate LEA protein/osmoprotectant synthase genes [21]. NAC TFs (e.g., ANAC019) induced by ABA/stress regulate cell wall modification and ROS scavenging genes [20]. Epigenetic modifications (e.g., histone H3K9 acetylation) also fine-tune expression—e.g., salt stress boosts SOS1 transcription via this modification [4].

### 2.3. Antioxidant System Activation

Stress-induced ROS (O_2_^−^, H_2_O_2_) cause cellular damage, so plants activate both enzymatic and non-enzymatic antioxidant systems. The enzymatic antioxidant system includes superoxide dismutase (SOD), ascorbate peroxidase (APX), Catalase (CAT) and glutathione reductase (GR), etc. [22,23,24,25]. The non-enzymatic antioxidant system includes ascorbic acid (AsA), glutathione (GSH), and carotenoids [22,23,24,25]. For example, drought-stressed wheat upregulates wheat superoxide dismutase (TaSOD) and wheat ascorbate peroxidase (TaAPX) to maintain ROS balance [3]; the ABA-SnRK2 signaling pathway further coordinates this system by upregulating antioxidant enzyme genes [20].

These pathways form a robust network, but high stress (e.g., severe salt overwhelming SOS pathway) limits their efficacy [21]—creating opportunities for microorganisms to intervene by targeting key nodes, as discussed later.

## 3. Advances in the Study of Microbial Strategies Against Drought and Salt Stresses in Plants

Research on microbial-mediated mitigation of plant drought and salt stress emerged in the late 20th century [26] and advanced rapidly in the 21st century [27,28,29]. This field covers diverse microbial functions, enabling systematic insights into underlying mechanisms. We summarized ~50 microorganisms involved in regulating plant stress resistance under drought and salt stress.

To ensure comprehensiveness of the literature collection, we conducted searches on core academic databases including Web of Science and ScienceDirect. The search was performed using keywords related to adversity types (e.g., “drought stress,” “salt stress”) and microbial species (e.g., “plant-growth-promoting rhizobacteria,” “arbuscular mycorrhizal fungi”), with no restrictions on authors, publication years, or journal types. Articles with content irrelevant to the research theme were excluded during the screening process. The final search primarily covered studies published in the past 30 years, resulting in the identification of approximately 1200 articles focusing on the interactions between microbes and plant stress responses. These studies mainly address key research areas such as plant oxidative stress regulatory mechanisms, plant–microbe interaction dynamics, maintenance of plant hormone homeostasis, enhancement of plant stress tolerance, microbial colonization strategies, and modulation of plant growth processes under stress conditions.

### 3.1. Advances in Microorganisms Involved in Plant Drought Stress Response

Global warming and frequent droughts increase drought stress incidence, causing plant physiological water deficit, even death, and global crop yield declines [30]. Plants can adapt to aridity by enhancing soil microbial ecosystems via morphological, molecular, physiological, and biochemical adjustments [6,31].

Under drought stress, microorganisms mitigate harm mainly through the following mechanisms: (1) Synthesizing growth-promoting substances—drought-tolerant microorganisms thicken plant cell walls, induce dormancy, accumulate osmotic regulators, and produce EPS to meet plant nutritional needs and improve survival [32]; *Bacillus cereus* L90 secretes cytokinins (CTKs) to promote stomatal opening, enhance photosynthesis, and reduce chlorophyll loss in walnuts [33]; nitrogen-fixing bacteria produce indole-3-acetic acid (IAA) to boost wheat root development and nutrient/water absorption [13]; *Bacillus megaterium* BOFC15 excretes spermidine (SPD) to increase ABA content and drought tolerance in *Arabidopsis thaliana* [34]; 1-amino-cyclopropane-1-carboxylate (ACC) deaminase-producing microorganisms can moderately reduce ethylene, balance stomatal opening and closing, while also slowing leaf senescence and extending the plant’s photosynthetic cycle [35]. (2) Enhancing nutrient/water uptake—inoculation with AZP2 strains elongates pistachio root hairs and induces lateral roots, expanding uptake area for water and minerals [36]; long-term changes in microbial communities (driven by root exudate shifts) promote nutrient mineralization, aiding plant drought recovery [37]. (3) Improving antioxidant ability—soybeans inoculated with *Penicillium anisopliae* show reduced lipid peroxidation and linolenic acid (LNA) accumulation, plus increased APX, peroxidase (POX), CAT, and SOD activities [32]. Table 1 provides a comprehensive overview of microbial effects on plants under drought stress.

### 3.2. Advancements in Microorganisms Involved in Plant Salt Stress Response

Salt stress causes high osmotic pressure, leading to plant water loss, cell death, and growth/yield inhibition [45]; however, plant–microbe symbiosis can promote plant growth in saline soils [46]. Key microbial mitigation strategies under salt stress include the following: (1) Regulating ion balance—*Bacillus velezensis* JC-K3 reduces Na^+^ uptake in wheat shoots, increases soluble sugars and chlorophyll, and alters endophytic/rhizosphere microbial diversity [14]; *Pseudomonas geniculate* MF-84 decreases maize Na^+^ uptake while increasing root K^+^/Ca^2+^ absorption, alleviating ionic toxicity [47]. (2) Enhancing nutrient absorption—arbuscular mycorrhizal fungi (*AMF*) form symbioses with plants to absorb diverse minerals, compensating for nutrient deficiencies [48]; *Trichoderma harzianum* (TH) increases S, Mn, Mg, Ca, and K contents in salt-stressed Indian mustard (by 7.0–36.3%) and reduces salt uptake [28]. (3) Modulating phytohormones and metabolites—bacteria with ACC deaminase reduce salt-induced ethylene (ETH) synthesis to enhance salt tolerance [49,50]; PGPR strains promote proline accumulation and POX activity in soybeans [51]; *Avicennia marina* bacteria secrete EPS to alleviate salt stress, solubilize inorganic phosphate, and improve nutrient uptake in rice [52]. (4) Activating plant signaling—endophytic bacteria in high-salinity soils induce plant synthesis of H_2_O_2_, salicylic acid (SA), and jasmonic acid (JA), upregulating sesquiterpene biosynthesis genes [53,54]; bacterial exocrines (IAA, ACCD deaminase) trigger plant regulatory pathways [16]. Table 2 provides a comprehensive overview of microbial effects on plants under salt stress. These microorganisms assist plants in coping with salt stress via multiple pathways.

In terms of microbial response mechanisms, the two stresses share commonalities. Both can regulate the osmotic pressure of plant cells to cope with osmotic stress by synthesizing osmotic regulators such as EPS. Additionally, both enhance plants’ nutrient uptake to support growth. However, differences exist: under drought stress, microorganisms focus on producing growth-promoting substances (e.g., secreting CTK and IAA) to regulate plant physiological processes; under salt stress, microorganisms prioritize ion balance regulation, reducing plants’ Na^+^ uptake while increasing the absorption of beneficial ions like K^+^ and Ca^2+^.

## 4. Mechanisms Behind Microbial Regulation of Plant Stress

**Figure 1:**
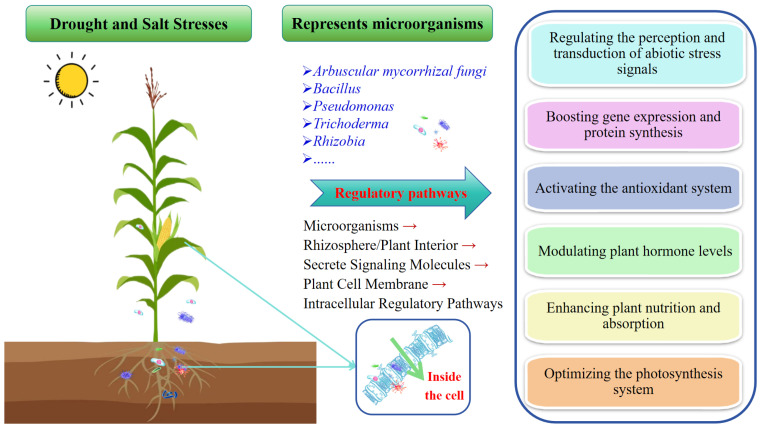
Schematic diagram illustrating the mechanisms of microbial regulation on plant abiotic stress responses. Note: Microorganisms secrete signaling molecules in the rhizosphere or inside plants, which act on plant intracellular regulatory pathways to alleviate drought and salt stresses. Red arrows indicate a process chain.

After comprehensively summarizing the advancements in the field of microorganisms aimed at enhancing plant stress tolerance under the aforementioned drought stress and salt stress conditions, six distinct mechanisms of action have been proposed. These mechanisms include the following: (1) regulating signal perception and transduction, (2) enhancing gene expression and protein synthesis, (3) activating the antioxidant system, (4) modulating phytohormone concentrations, (5) enhancing plant nutrition and absorption capabilities, and (6) optimizing the photosynthetic system. These mechanisms are schematically depicted in Figure 1.

### 4.1. Regulating the Perception and Transduction of Abiotic Stress Signals to Enhance Plant Adaptive Responses

Plants possess sophisticated sensing, signaling, and response mechanisms to cope with external stressors [20,21]. Chemical signaling between plants and microorganisms is a collaborative outcome of their interactions [66]. Notably, most key regulators involved in plant defense responses operate within a complex network of modulators rather than following a linear pathway [67]. Prolonged exposure to abiotic stress can induce adaptive changes in specific microbial subgroups, enabling them to maintain efficient routine metabolic functions even under stressful conditions [68]. This adaptation leads to the emergence of microorganisms with enhanced stress tolerance, which in turn promotes crop growth. During plant responses to external stress, root exudates serve as a critical communication bridge between plants and microorganisms: they provide essential energy for microbial growth and facilitate direct interactions via specific signaling molecules [37]. Importantly, the composition and quantity of root exudates dynamically change throughout plant development [69], and stress factors further alter their properties and production [70]. Microorganisms can recognize root exudates, perceive stress signals, and recruit additional beneficial microbial populations to perform functional roles that benefit the host plant—fostering a symbiotic relationship that supports the mutual survival and development of both parties [71]. For example, a recent study demonstrated that under salt stress, wild soybeans secrete purine analogs as signaling molecules; upon recognition by *Pseudomonas aeruginosa*, these bacteria secrete secondary signaling substances to inversely regulate the plant’s stress signal transduction pathway, ultimately enhancing plant salt tolerance [72]. Specifically, the secondary signaling substances secreted by *Pseudomonas aeruginosa* bind to plant root cell membrane receptors, activating the plant’s MAPK signaling pathway and further regulating the expression of stress-responsive genes (e.g., *RD29A*), thereby linking microbial signals to plant stress regulatory pathways.

### 4.2. Boosting Gene Expression and Protein Synthesis for Overall Plant Metabolic Regulation

Gene regulatory strategies offer innovative methodologies for cultivating crops that demonstrate tolerance to abiotic stress conditions [73]. In response to abiotic stress environments, plants have naturally developed diverse mechanisms and regulatory networks that modulate the expression patterns of their genes, enabling them to adapt and thrive. Additionally, these regulatory systems facilitate interactions between plants and microorganisms, further enhancing their survival capabilities [74]. Firstly, the expression of endogenous genes can be modulated under stressful conditions, resulting in either upregulation or repression. For instance, *Pseudomonas fluorescens* enhances the expression of the ACC deaminase gene acdS, thereby reducing the concentration of ETH and improving resistance to abiotic stress [75]. Similarly, tomato plants inoculated with TH exhibit significant elevations in transcript levels of *NAC1*, dehydrin *TAS14*, and *P5CS* genes [76]. Additionally, abscisic acid-induced *myb1* (SlAIM1) [77] and stress-inducible genes such as *CaACCO* and *CaLTPI* are regulated in a comparable manner [78]. Additionally, endophytes harbor genes crucial for biological nitrogen fixation, a process that facilitates the conversion of atmospheric nitrogen (N_2_) into ammonium and nitrate within the host plant [79]. Finally, it promotes the expression of the drought-induced gene wzy2 in wheat, thereby upregulating the expression of drought-related proteins and enhancing the dehydration tolerance and drought stress adaptability of two winter wheat varieties [39].

### 4.3. Activating the Antioxidant System to Strengthen Plant Tolerance

In plants, ROS play a pivotal role in regulating a broad spectrum of biological processes, such as cell signaling, growth, and development [22,23]. Nevertheless, when ROS concentrations exceed a critical threshold—often triggered by abiotic stresses like drought and salt—they induce oxidative damage to cellular components, including lipids, proteins, and nucleic acids [22]. Notably, abiotic stress typically leads to a marked surge in plant ROS levels, which in turn inhibits growth and development by disrupting metabolic homeostasis [24]. Beneficial microorganisms sustain plant antioxidant system functionality to mitigate stress-induced oxidative damage via two mechanisms: (1) directly secreting extracellular antioxidants (e.g., superoxide dismutase, SOD) to scavenge rhizospheric or apoplastic ROS; (2) indirectly enhancing plant endogenous antioxidant synthesis by activating stress-responsive signaling pathways (e.g., salicylic acid-mediated pathways) or upregulating antioxidant-related genes [25]. This dual regulation boosts the accumulation of both enzymatic antioxidants (e.g., SOD, CAT, peroxidase (POD)) and non-enzymatic antioxidants (e.g., glutathione (GSH), AsA, tocopherols, and carotenoids), effectively shielding plants from ROS-induced cellular damage [80]. For instance, inoculation with Penicillium anisopliae has been shown to reduce lipid peroxidation and enhance SOD, CAT, and ascorbate peroxidase (APX) activities in soybean under drought stress, mirroring this protective mechanism [32]. PGPB further reinforces antioxidant defense. Specifically, PGPB enhance both enzymatic and non-enzymatic antioxidant pathways, which collectively preserve cellular redox homeostasis, elevate ROS scavenging capacity, and optimize biosignature traits (e.g., nutrient uptake efficiency, photosynthetic stability). These effects ultimately translate to improved tolerance of plants to drought and salt stresses [44].

### 4.4. Modulating Plant Hormone Levels to Stimulate Growth in Response to Adversity

Phytohormones act as pivotal signaling molecules in plant responses to environmental stresses [81]. During interactions with microorganisms, plants can mitigate stress effects through hormone-mediated pathways, involving the regulation of IAA, ABA, ETH, SA, and JA [82]. This regulatory process operates through three primary mechanisms: First, symbiotic microorganisms either produce phytohormones or their analogs or induce plants to secrete these signaling molecules [83]. For example, several bacterial genera including *Pseudomonas*, *Pantoea*, *Hymenobacter*, and *Rhizobium* synthesize IAA analogs [84]. Similarly, TH produces metabolites such as hazylactone and 6-pentyl-α-pyrone that mimic IAA activity, effectively stimulating plant growth [85]. Conversely, *Variovorax* species modulate root development by regulating IAA metabolism—specifically targeting key enzymes in the IAA pathway through unique degradation sites to adjust hormone levels, thereby influencing root cell division, elongation, and differentiation [66]. Second, microorganisms significantly impact plant hormone metabolism and synthesis. A well-documented example is the reduction in ethylene levels by PGPR through ACC deaminase activity. This enzyme catalyzes the conversion of ACC—the immediate precursor of ethylene—into ammonia and α-ketobutyric acid [35,75,86]. Similar ethylene-modulating effects have been observed in *Arthrobacter* and *Bacillus* species [78]. Fungal inoculation, meanwhile, typically reduces ABA levels while altering SA and JA concentrations [87]. Additionally, *Bacillus* licheniformis SA03 activates ABA-dependent nitric oxide (NO) synthesis, thereby enhancing the stress resistance of chrysanthemums. As a secondary messenger, NO further improves chrysanthemums’ stress tolerance by regulating downstream stress-responsive pathways [88]. Specifically, NO functions as a signaling hub by mediating S-nitrosylation of target proteins, regulating components of signaling pathways (e.g., MAPK, cGMP), and maintaining ROS homeostasis. Through these mechanisms, it coordinates with plant hormones in a synergistic or antagonistic manner to regulate plant growth, development, and stress responses, with its regulatory effects exhibiting concentration dependence. Notably, NO also serves as a pivotal signaling molecule in plant–microbe interactions: it mediates the regulatory role of microorganisms in plant stress tolerance and participates in the crosstalk between microorganisms and plant hormones [89]. Third, microorganisms influence hormone transport and functional mechanisms within plants. *Pseudomonas malodorata*, for instance, modifies endogenous hormone accumulation in roots and shoots, increasing levels of gibberellins (GA), IAA, CTK, and ABA to mitigate stress impacts [90]. PGPR enhance wheat tolerance to abiotic stresses through a coordinated hormonal strategy: increasing IAA levels, reducing ABA and ACC concentrations, and regulating the expression of ethylene signaling pathway components (e.g., CTR1) and DREB2 transcription factors [41].

### 4.5. Enhancing Plant Nutrition and Absorption to Improve Tolerance

The rhizosphere is a crucial habitat for microorganisms, where root exudates attract microbial colonization and provide nutrients essential for microbial proliferation [68,69,91]. *Mycorrhizal fungi* (MF) play a pivotal role in facilitating host water acquisition under stress and stimulating lateral root formation, thereby increasing root surface area and promoting root uptake [92]. Additionally, radial mycelium extension induced by *AMF* inoculation effectively enhances nutrient uptake [93]. Mycelium forms a filamentous network via apical extension and subapical branching; its rapid apical elongation enables insertion into the plant cell wall [94], which facilitates the regulation of osmotic balance and composition. Mycorrhizal symbiosis significantly improves water retention, thereby alleviating dehydration and metabolic disorders [95]. Furthermore, microbial fertilizers can modulate the concentration of IAA, effectively reducing root sensitivity and promoting root elongation and growth [96].

Roots release chemical signals and nutrients that influence the rhizosphere microbial community [97]. *Pseudomonas*, *Rhizobium*, *Bacillus*, *Penicillium*, and *Aspergillus* are well-recognized efficient phosphate-solubilizing microorganisms (PSMs) [59]. These microbes secrete organic acids to lower the surrounding soil pH, thereby solubilizing phosphorus from mineral phosphates for subsequent plant absorption [98]. Additionally, soil phosphorus-solubilizing bacteria (PSB) not only prevent phosphorus fixation but also enrich the soil with micronutrients [99]. Mycorrhizal colonization further enhances plant P uptake [42], and phosphatases secreted by *AMF* and plant root colonization collectively improve plant P absorption efficiency [27]. Elevated ACC deaminase activity is associated with increased accumulation of nutrients such as N, P, K, and other essential elements, thereby promoting root growth and colonization [38]. Nitrogen-fixing bacteria (NFB) assist plants in amino acid synthesis by converting atmospheric nitrogen and regulating its utilization and absorption, thereby enhancing plant nutritional capacity [12]. A variety of metabolic compounds produced by plant-associated microorganisms—including growth-promoting substances—play key roles in rhizosphere function and indirectly enhance plant stress resistance. Among these compounds, ACC deaminase, IAA, siderophores, and organic acids are particularly pivotal [38].

### 4.6. Optimizing the Photosynthesis System to Promote the Synthesis of Essential Substances, Safeguarding Plant Growth and Development Amidst Adversity

Exogenous microorganisms can effectively regulate plant photosynthetic and alleviate stress-induced adverse effects. The most intuitive manifestation is increased photosynthetic pigment content in leaves. By enhancing water uptake efficiency and scavenging ROS, exogenous microorganisms improve leaf physiological activity, thereby significantly increasing plant photosynthetic rates [38]. Additionally, microorganisms maintain the dynamic balance between plant water status and photosynthesis by regulating stomatal conductance, reducing negative impacts from excessive transpiration—an effect *Trichoderma* exerts on various crops [6].

Acinetobacter calcoaceticus strain X128 secretes CTK, a hormone that promotes stomatal opening and inhibits chlorophyll degradation. This keeps photosynthetic limitations at the reversible stomatal level, delaying irreversible mesophyll cell damage from non-stomatal limitations and enhancing post-rehydration recovery of photosynthetic function [100]. *AMF* alleviate drought-induced inhibition of photosynthetic apparatus in wheat by maintaining PSII stability, improving plant water status (measured via RWC), and optimizing nutrient supply—ultimately increasing biomass [101]. For instance, *Acinetobacter calcoaceticus* X128-secreted CTK not only maintains stomatal opening but also upregulates the expression of chloroplast-related genes (e.g., *rbcL*, which encodes Rubisco large subunit), thereby protecting chloroplast structure and function. However, the direct mechanisms by which microorganisms regulate chloroplast function require further in-depth research.

Under salt stress, tomato plants inoculated with wild-type endophytes (*Pseudomonas fluorescens* YS56, *Pseudomonas migulae* 8R6) containing ACC deaminase exhibit significantly reduced endogenous ethylene levels, delayed chlorophyll decomposition, and maintained photosynthetic capacity [58]. Notably, *AMF* reduces Na^+^ toxicity through selective K^+^ uptake, while PGPR binds Na^+^ via EPS secretion—reducing Na^+^ transport to leaves. These actions preserve the activity of key enzymes (e.g., Rubisco) and protein synthesis processes [27,51].

## 5. Conclusions and Outlook

This review comprehensively summarizes the latest research progress (1994–2025) on the role of core microbial groups—including PGPR, *AMF*, and endophytes—in enhancing plant tolerance to drought and salt stresses. It further synthesizes and proposes six core mechanisms by which microorganisms improve plant stress resistance: (1) modulation of stress signal perception and transduction; (2) enhancement of gene expression and protein biosynthesis; (3) activation of antioxidant defense systems; (4) regulation of phytohormone homeostasis; (5) promotion of plant nutrient status and absorption efficiency; and (6) optimization of photosynthetic processes.

While this review outlines the six core mechanisms of microbial-mediated stress alleviation, critical gaps and inconsistencies in existing research must be acknowledged. First, microbial treatments do not uniformly enhance plant drought or salt tolerance. For example, certain PGPR strains (e.g., *Bacillus subtilis* strain GB03) significantly improve maize drought tolerance but show no obvious effect on wheat under the same stress intensity [102,103]; similarly, *AMF* colonization (e.g., *Rhizophagus irregularis*) enhances salt tolerance in tomato cultivar ‘Moneymaker’, yet fails to alleviate salt damage in ‘Ailsa Craig’ [104,105]. This variability is linked to four key factors: plant genotype (differences in root exudate composition and stress response pathways among species/cultivars affect microbial colonization and function), soil properties (acidic soils with low organic matter inhibit PGPR growth, while high bulk density impedes *AMF* hyphal extension), stress severity (mild salt stress stimulates microbial metabolite secretion, but extreme drought/high-salt conditions impair microbial viability), and microbial strain specificity (different strains of the same genus have distinct capacities for hormone production or osmolyte synthesis).

Second, the direct interaction mechanisms between microorganisms and plant chloroplast function remain unclear—such as how microbial signaling molecules regulate chloroplast-related gene expression or maintain photosystem II stability under stress. Additionally, lab-scale results lack systematic field validation, as most laboratory experiments rely on controlled conditions inconsistent with real agricultural scenarios. Notably, the lab-field gap is pronounced: laboratory tests use homogenized soil substrates, constant temperature/humidity, and single-stress treatments, which fail to replicate natural environmental complexity, including soil variability (e.g., heterogeneous texture, uneven nutrient distribution, fluctuating pH in field soils) and unstable microbial durability (microbial colonization ability and functional activity are reduced by extreme field conditions like sudden temperature changes or soil compaction). These limitations undermine the reliability of extrapolating lab findings to real-world agricultural production.

To address these gaps and inconsistencies, future research should prioritize the following: (1) Strengthening field validation under complex stress scenarios (e.g., “drought-salt combined stress” or “salt stress with soil degradation”) by quantifying agronomic traits (e.g., yield, water use efficiency) and microbial functional stability (e.g., enzyme activity, metabolite secretion) to bridge the lab-field gap. (2) Deciphering tripartite interactions (microbes–plants–environment/agronomic factors) to optimize microbial colonization—for example, exploring how adjusting nitrogen fertilizer rates enhances *AMF* colonization in wheat roots, or developing microbial consortia (combining stress-tolerant PGPR and *AMF*) to reduce strain-specific variability. (3) Tailoring rhizosphere environments (via optimized irrigation, nutrient supply, or organic amendments like biochar) to promote beneficial microbial recruitment and proliferation, mitigating adverse soil-induced inhibition. (4) Expanding research on microbial metabolites (e.g., exopolysaccharides, cyclic peptides) to evaluate their roles in plant growth promotion and stress alleviation, plus synergistic application with low-dose pesticides/fertilizers. (5) Advancing interdisciplinary collaboration (integrating agriculture, microbiology, soil science, and molecular biology) to exploit microbial resources—such as using metagenomics to identify key stress-tolerant taxa in saline–alkali soils or engineering microbes with enhanced stress adaptability—providing practical support for sustainable agriculture and ecological restoration.

## Figures and Tables

**Table 1:** Effects of microorganisms on plants under drought stress.

Name of Microorganism	Test Crop	Level of Stress	Microorganism Dosage Usage	Response of Microbial Inoculated Plants to Drought Stress as Compared to Controls	Mechanism of Action	Reference
Arbuscular mycorrhizal fungi (*AMF*)	Maize (*Zea mays* L.)	35%, 55%, 80% field water holding capacity	10 mL 10^8^ CFU/mL	Enhances root colonization, water utilization and root hydraulic conductivity, thereby improving nutrient uptake in the corn root system and aboveground	(3) (4) (5) (6)	[38]
*Chaetomium globosum ND35*	Wheat (*Triticum aestivum* L.)	30% of maximum water holding capacity	10^6^ CFU/mL	Promote root and plant development during the seedling stage of winter wheat, allowing wheat to enter the three-leaf stage earlier, enhance drought avoidance, and at the same time improve root activity and increase drought resistance	(2) (3) (5)	[39]
*Glomous mosseae*	Chinese wildrye (*Leymus chinensis*)	10% PEG	5 g *AMF* strain	Inhibition of Na^+^, Cl^−^ uptake, enhancement of Ka^+^ uptake, elevated proline content, elevated antioxidant defense enzyme content	(1) (3) (5)	[35]
*Azospirillum lipoferum* AZ1, *Azospirillum lipoferum* AZ45, *Azospirillum lipoferum* AZ9	Wheat (*Triticum aestivum* L.)	80, 50, 25% field water holding capacity	3.2 × 10^9^ CFU/mL	Indole-3-acetic acid (IAA), and proteins, polyamines, nitrogen fixation, root growth promotion	(1) (4) (5)	[40]
*Bacillus cereus* L90	Walnut (*Juglans regia* L.)	Water content 34.64%	2 × 10^8^ CFU/mL	Promotes secretion of cytokinin (CTK), which increases net photosynthetic rate, stomatal conductance, intercellular CO_2_ concentration and chlorophyll content	(1) (3) (4) (6)	[33]
*Bacillus megatherium* BOFC15	Arabidopsis thaliana (*Arabidopsis thaliana* L. Heynh.)	200 min of dehydration	1 mL bacterial diluent	Increase plant biomass, improved root structure, and enhanced photosynthetic capacity.	(1) (2) (3) (4) (6)	[34]
*Sarthrobacter protophormiae* SA3 *Dietzia natronolimnaea* STR1 *Bacillus subtilis* LDR2	Wheat (*Triticum aestivum* L.)	10% PEG	25 mL 10^5^ CFU/mL	Increase IAA content, photosynthetic efficiency, reduced abscisic acid and Enzyme 1-amino-cyclopropane-1-carboxylate (ACC) content	(1) (2) (4) (6)	[41]
*AMF*	Spring wheat (*Triticum aestivum* L.)	40% soil moisture content	Inoculums (1600 propagules/g) were mixed with wheat seeds at 10 mL/kg pre-wetting rate.	Increase N and P concentrations in stems and grains resulted in a significant increase in the plant’s water use efficiency	(1) (5) (6)	[42]
*Pseudomonas fluorescens* strains FB-49	Acacia martius (*Acacia farnesiana* L. Willd.)	Keep 20% water content	15 mL 10^8^ CFU/mL	Increase root length, aboveground node length and dry biomass of plants	(1) (5)	[43]
*Azotobacter brazilensis* *Bacillus* sp.	Tropical trees (*Pinus tropicalis* Morelet)	14, 30% humidity	50 mL 10^6^ CFU/mL	Induce greater accumulation of secondary compounds and increased leaf area.	(1) (3)	[44]

Note: (1) regulating the perception and transduction of abiotic stress signals to enhance plant adaptive responses; (2) boosting gene expression and protein synthesis for overall plant metabolic regulation; (3) activating the antioxidant system to strengthen plant tolerance; (4) modulating plant hormone levels to stimulate growth in response to adversity; (5) enhancing plant nutrition and absorption to improve resilience; (6) optimizing the photosynthesis system to promote the synthesis of essential substances, safeguarding plant growth and development amidst adversity.

**Table 2:** Effects of microorganisms on plants under salt stress.

Name of Microorganism	Test Crop	Level of Stress	Microorganism Dosage Usage	Response of Microbial Inoculated Plants to High Salt Stress as Compared to Controls	Mechanism of Action	Reference
*Bacillus subtilis* NCD-2	Tomato (*Solanum lycopersicum* L.)	100 mmol/L NaCl	1.0 × 10^9^ CFU/mL	Enhanced resistance enzyme activity, increased ABA content, and enriched rhizosphere beneficial microbes	(1) (3) (4)	[55]
*Brevibacterium* *sediminis* Strain IBGE3C	Rice (*Oryza sativa* L.)	0.2–1.2% NaCl	Seed soaking	Improve rice varieties with different levels of salt tolerance	(1) (3) (4)	[56]
*Burkholderia phytofirmans* PsJN *Enterobacter* sp. FD17	Maize (*Zea mays* L.)	25 mmol/L NaCl	Mix 20 mL of bacterial suspension with 100 g of sterilized peat	Reduce xylem Na^+^ concentration uptake, thereby maintaining nutrient balance and promoting plant growth	(1) (4) (5)	[57]
*Pseudomonas fluorescens* YsS6 *Pseudomonas migulae* 8R6	Tomato (*Solanum lycopersicum* L.)	165, 185 mmol/L NaCl	1.75 × 10^8^–1.97 × 10^8^ CFU/ml	Higher fresh and dried biomass, higher chlorophyll content and more flowers and buds reduce salt stress	(1) (4) (6)	[58]
*Klebsiella pseudomonas* *Agrobacterium ochrobactrum*	Peanut (*Arachis hypogaea* L.)	4, 8% NaCl	10^8^ cells/mL	Increase solubilization of phosphorus; promotes stem length, root length, shoot and root growth in peanut plants	(1) (2) (3) (4) (5)	[59]
*Bacillus subtilis* 10-4	Babury Wolfberry Fruit (*Lycium chinense* Miller)	2% NaCl	10^5^ CFU/mL	Inhibition of Salicylic acid (SA) accumulation, increase in water storage capacity in leaf tissues	(1) (3) (4) (5)	[60]
*Bacillus amyloliquefaciens* RWL-1	Rice (*Oryza sativa* L.)	40 g/L NaCl	10^8^ CFU/mL	Increase essential amino acids and SA, decreased ABA levels	(1) (4) (5)	[61]
*Bacillus cereous* Pb25	Mung bean (*Vigna radiata* (L.) R. Wilczek)	Electric conductivity 9 dS/m	10^7^–10^8^ CFU/mL	Increase plant antioxidant enzyme activity, proline, potassium, nitrogen, and phosphorus accumulation; decreased sodium accumulation	(1) (3) (4) (5)	[62]
*Staphylococcus equorum* strain EN21	Tomato (*Solanum lycopersicum* L.)	30% NaCl	10^9^ CFU/mL	Increase seed vigor index, branch length and root dry weight of plants	(1) (3) (4) (5) (6)	[63]
*Pseudomonas* strains AK-1 *Bacillus* strains SJ-5	Soybean (*Glycine max* (L.) Merr.)	100 mmol/L NaCl	10^8^ CFU/mL	Increase plant biomass, leaf water content, photosynthetic activity; increased proline accumulation and peroxidase (POX) activity	(1) (2) (3) (4) (5) (6)	[51]
*Avicennia marina*	Rice (*Oryza sativa* L.)	0.5–22.5% NaCl	10^8^ CFU/mL	Promotes solubilization of inorganic phosphate and enhances nutrient uptake	(1)	[52]
*Piriformospora indica*	Barley (*Hordeum vulgare* L.)	100, 300 mmol/L NaCl	The mycelial colonization rate is 50–60%	Enhancement of Ascorbate peroxidase (APX) activity in barley roots	(1) (3) (4) (6)	[64]
*Pseudomonas* strains PF1/TDK1	Rice (*Oryza sativa* L.)	100 mmol/L NaCl	0.2 g/L	Plant height, root length, aboveground and root dry weight were significantly increased	(1) (3) (4) (6)	[65]
*Trichoderma harzianum*	Indian mustard (*Brassica juncea* L.)	100, 200 mmol/L NaCl	2 × 10^9^ CFU/mL	Increase oil content improves absorption of essential nutrients, enhances antioxidant and osmotic agent accumulation, and reduces salt absorption	(1) (3) (4) (5) (6)	[28]

Note: (1) regulating the perception and transduction of abiotic stress signals to enhance plant adaptive responses; (2) boosting gene expression and protein synthesis for overall plant metabolic regulation; (3) activating the antioxidant system to strengthen plant tolerance; (4) modulating plant hormone levels to stimulate growth in response to adversity; (5) enhancing plant nutrition and absorption to improve resilience; (6) optimizing the photosynthesis system to promote the synthesis of essential substances, safeguarding plant growth and development amidst adversity.

## Data Availability

No new data were created or analyzed in this study. Data sharing is not applicable to this article.
